# An Iron 13*S*-Lipoxygenase with an α-Linolenic Acid Specific Hydroperoxidase Activity from *Fusarium oxysporum*

**DOI:** 10.1371/journal.pone.0064919

**Published:** 2013-05-31

**Authors:** Florian Brodhun, Alvaro Cristobal-Sarramian, Sebastian Zabel, Julia Newie, Mats Hamberg, Ivo Feussner

**Affiliations:** 1 Georg-August-University, Albrecht-von-Haller-Institute for Plant Sciences, Department of Plant Biochemistry, Goettingen, Germany; 2 Karolinska Institute, Institute for Medical Biochemistry and Biophysics (MBB), Stockholm, Sweden; Soonchunhyang University, Republic of Korea

## Abstract

Jasmonates constitute a family of lipid-derived signaling molecules that are abundant in higher plants. The biosynthetic pathway leading to plant jasmonates is initiated by 13-lipoxygenase-catalyzed oxygenation of α-linolenic acid into its 13-hydroperoxide derivative. A number of plant pathogenic fungi (e.g. *Fusarium oxysporum*) are also capable of producing jasmonates, however, by a yet unknown biosynthetic pathway. In a search for lipoxygenase in *F. oxysporum*, a reverse genetic approach was used and one of two from the genome predicted lipoxygenases (FoxLOX) was cloned. The enzyme was heterologously expressed in *E. coli*, purified via affinity chromatography, and its reaction mechanism characterized. FoxLOX was found to be a non-heme iron lipoxygenase, which oxidizes C_18_-polyunsaturated fatty acids to 13*S*-hydroperoxy derivatives by an antarafacial reaction mechanism where the bis-allylic hydrogen abstraction is the rate-limiting step. With α-linolenic acid as substrate FoxLOX was found to exhibit a multifunctional activity, because the hydroperoxy derivatives formed are further converted to dihydroxy-, keto-, and epoxy alcohol derivatives.

## Introduction

Lipoxygenases (LOXes) are non-heme iron or manganese containing enzymes that are abundant in mammals [1] and plants [2], but have also been described in some fungi [3], [4] and bacteria [5]. They typically catalyze the regio- and stereospecific oxidation of polyunsaturated fatty acids containing a 1*Z*,4*Z*-pentadiene system to hydroperoxy derivatives [6]. These compounds act as precursors for further enzymatic cascades leading to a large variety of signal molecules that are named oxylipins [3], [5], [7]. Due to the important signaling functions of LOX-derived products in plants and mammals these enzymes have been intensively studied over the past decades. Crystallographic studies demonstrated that despite significant differences in size, mammalian and plant LOXes have a highly similar over-all structure [1]: The N-terminal region is made up by a domain that consists mainly of β-sheets forming a barrel-like structure. This so-called β-barrel domain resembles those of C2-domains known from peripheral membrane proteins where they are functioning as membrane binding sites [8]. The C-terminal region on the other hand forms the mainly α-helical catalytic domain bearing the mono-nuclear non-heme iron [1] which is, in case of plant LOXes, coordinated by the N-atoms of three conserved His-residues, the O-atom of one particular Asp and the carboxy terminus of the C-terminal Ile [9]. In contrast, fungal LOXes have so far been described to contain manganese rather than iron in their active site [3]. The central and rate-limiting step of the LOX reaction is the stereospecific hydrogen abstraction from the bis-allylic C-atom of the pentadiene system. The resulting carbon centered radical is delocalized via the π- electron system and can be attacked by molecular oxygen at three theoretical positions, i.e. C-1, C-5 or C-3 [6]. With 18∶2(n-6) as substrate for LOX reaction, oxygen insertion can thus take place either at the C-9, C-11 or C-13 positions of the fatty acid backbone, forming the respective 9-, 11- or 13-hydroperoxy derivatives. While oxygen insertion at C-1 and C-5, yielding mono-allylic hydroperoxides, is typically found with mammalian and plant LOXes, formation of C-3-centered bis-allylic hydroperoxides has been observed only in very few cases [10], [11]. The comparatively unstable bis-allylic hydroperoxides have been identified as products of fungal manganese LOX (13*R*-MnLOX) which was isolated as native protein from the fungus *Gaeumannomyces graminis* [12]. In later studies 13*R*-MnLOX was cloned, heterologously expressed in *Pichia pastoris* [13], and characterized in-depth, demonstrating that this enzyme differs from classical LOXes in at least three aspects: (i) instead of iron 13*R*-MnLOX contains manganese [14], [15], (ii) it does not catalyze an antarafacial but a suprafacial reaction [11], [16], (iii) the enzyme forms bis-allylic hydroperoxy fatty acids which can be isomerized yielding the respective 13*R*-hydroperoxy fatty acid derivatives with C_18_-polyunsaturated fatty acids as substrates [11], [17]. Site-directed mutagenesis was used not only to identify residues involved in coordinating the catalytic metal but also to identify amino acids influencing catalysis and product formation [13], [18], [19], [20]. During preparation of the present manuscript, a 9*S*-MnLOX was the identified and characterized [21]. This enzyme showed a high sequence identity (77%) towards 13*R*-MnLOX and exhibited similar mechanistic features. It should be pointed out, that until now the two MnLOXes are the only ascomycete LOXes that have been characterized by heterelogous expression. Only very recently the first study on the recombinant expression and characterization of a LOX from the Basidiomycete *Pleurotus sapidus* has been reported [22].

In addition to the metabolites formed by MnLOX further oxylipins have been identified in different fungal species [23]. On the one hand these data indicate that the structural diversity of fungal oxylipins is considerably high and that their biosynthetic pathways might differ significantly from those present in mammals or plants [3]. On the other hand, however, different studies demonstrated that some plant pathogenic fungi (e.g. *Fusarium oxysporum* and *Botryodiplodia*/*Lasiodiplodia theobromae*) are also capable of synthesizing plant specific oxylipins as for example jasmonic acid (JA) [24], [25], [26], [27]. Because this phytohormone functions as an essential signaling molecule regulating developmental processes as well as stress and defense responses in plants [28], these studies support the proposed role of fungal oxylipins as mimics of host metabolites [29]. A recent study on JA biosynthesis in *L. theobromae* indicated that fungal JA is derived from a fatty acid precursor and is formed via the intermediary formation of a cyclopentenone analogous to the plant pathway [26]. Here, JA biosynthesis starts with free α-linolenic acid (18∶3(n-3), *x*:*y*(n-*z*) denotes a fatty acid with *x* carbons and *y* double bonds in position *z* counting from the methyl end) that is oxidized by a 13*S*-LOX yielding the 13*S*-hydroperoxy derivative. This metabolite serves as a substrate for further sequential conversions that lead to the formation of JA. A subsequent study with *L. theobromae* aimed at identifying the dioxygenase responsible for the initial oxidation step in JA-biosynthetic pathway; however, the existence of plant-like JA-biosynthetic enzymes in *L. theobromae* could not be supported. In fact, instead an unusual 9*R*-dioxygenase that catalyzes the suprafacial oxidation of C_18_-polyunsaturated fatty acids to the corresponding 9*R*-hydroperoxy fatty acids was observed [30]. Since 9-hydroperoxy derivatives cannot be further transformed to JA, the biosynthetic route of fungal JA-biosynthesis remains elusive.

The objective of this study was to continue the analysis of the first step of fungal JA biosynthesis using a reverse genetic approach. As no sequence information of the *L. theobromae* genome is yet available, the project focused on *Fusarium oxysporum*. A BLAST search of the *F. oxysporum* genome revealed two sequences with homology to LOX. Sequence as well as phylogenetic analyses indicated that one of those sequences was similar to the previously described 13*R*-MnLOX being specific for fungi. Analysis of the second sequence suggested that it may encode a 13*S*-LOX similar to those found in the JA-biosynthetic pathway of plants. Therefore, this sequence was cloned, the protein recombinantly expressed in *E. coli*, purified by affinity chromatography, and the purified enzyme was characterized. The enzyme, here named FoxLOX, was found to act as a regular 13-LOX when 18∶2(n-6) was used as the substrate, however, surprisingly the JA precursor 18∶3(n-3) was metabolized both by dioxygenase and hydroperoxidase activities.

## Materials and Methods

### Materials

All chemicals used in this study were either from Sigma-Aldrich or from Carl Roth & Co. Agarose was purchased from Biozym Scientific GmbH while all fatty acids were from Cayman Chemicals. Acetonitrile was from Fisher Scientific and restriction enzymes were purchased from MBI Fermentas.

The following reference oxylipins were purchased from Lipidox Co. (Stockholm, Sweden) or Larodan (Malmö, Sweden): 13-HOT, 13-KOT, 12*S*,13*S*-epoxy-11*R*-hydroxy-9*Z*,15*Z*-octadecadienoic acid (*threo* form), 12*S*,13*S*-epoxy-11*S*-hydroxy-9*Z*,15*Z*-octadecadienoic acid (*erythr*o form), *erythro*- and *threo*-15,16-dihydroxyoctadecanoic acids, and 9,16-dihydroxyoctadecanoic acid.

### Cultivation and RNA-extraction

*Fusarium oxysporum* (*Fusarium oxysporum f. sp. lycopersici strain 4287*) was obtained from the Fungal Genetics Stock Center (Kansas City, USA) [31]. Mycelium was statically incubated in standard Potato Dextrose Broth (26.5 g/L) at 20°C for 1–5 weeks. The mycelium was harvested via filtration through cellulose membrane (90 mm pore size) and shock frozen using liquid nitrogen. For extraction of RNA the TRIZOL-method was used: Briefly, 500 mg of mycelium were disrupted in 2 mL TRIZOL (38% (v/v) Phenol, 0.8 M guanidinium chloride, 0.4 M ammonium thiocyanate, 133.6 mM sodium acetate, 5% glycerol) employing a glass Potter-Elvehjem homogenizer with Teflon coated pestle (from Wheaton, USA). 400 µL CHCl_3_ were added and the homogenate was centrifuged at 3200×g at 4°C for 30 min. The upper phase was incubated with 2 mL of phenol/methylenechloride and centrifuged at 3200×g at 4°C for 15 min. An equal amount of isopropanol was added to the upper phase and incubated at −20°C for 12 h. After centrifugation at 20,000×g and 4°C for 30 min, the precipitate was washed with 70% (v/v) ethanol and dried at room temperature. RNA was incubated at 65°C in RNAse-free water for 5 min and was stored at −80°C. For cDNA synthesis the RevertAid H Minus Reverse Transcriptase Kit from Fermentas was used according to the manufacturer’s instructions.

### Analysis of the Fatty Acid Composition

Prior to extraction fungal material was ground for 4 min to a small powder by employing a nitrogen-cooled beat mill (Retsch MM200) for 4 min with a frequency of 30 sec^−1^. The powder was stored at −80°C. Lipids were extracted as follows: 4 g of fungal powder was incubated with 20 mL CHCl_3_/MeOH (1/1, v/v) at 4°C for approx. 18 h. As internal standard 20 µg 17∶0 and 200 µg Tri-17∶0 were added. After separation of both phases the aqueous phase was re-extracted by incubation with 20 mL CHCl_3_ for 4 h at 4°C. Both organic phases were combined, dried under streaming nitrogen and re-dissolved in 800 µL MeOH. Lipid bound fatty acids were converted to corresponding methyl esters by transesterification that was performed in accordance to [32]. Briefly, the dried sample was resuspended in 330 µL toluene/methanol (2/1, v/v) and 170 µL sodium methoxide (0.5 M in methanol; Sigma-Aldrich), and incubated for 20 min at room temperature. 500 µL of saturated NaCl solution and 50 µL of 32% (v/v) HCl were added. The resulting methyl esters were extracted with hexane. The sample was dried under streaming nitrogen and re-dissolved in acetonitrile/water/acetic acid (50/50/0.1, v/v/v). Free fatty acids were methylated in 400 µL MeOH containing 6.5 µL of 2 M (diazomethyl)trimethylsilane (in hexane; Sigma-Aldrich) and incubated at room temperature for 30 min on a shaker. The reaction was terminated by adding 0.2 µL acetic acid. After evaporation of the solvent(s) under streaming nitrogen, the extract was redissolved in acetonitrile and analyzed as described as before [33].

### Cloning and Functional Expression of FoxLOX in *E. coli*

The FoxLOX gene (Broad Institute accession number FOXG_04807) was amplified from fungal cDNA using the ExTaq Polymerase Kit (TaKaRa Biotechnology) and the following primers containing the NheI and NotI recognition site, respectively: 5′-ACGGCTAGCATGGCAACAGAAGCTCCTTTAGCAC-3′ (sense) and 5′-ACGGCGGCCGCCTAAATCAAGATAGAAACCGCGG-3′ (antisense). The following PCR-conditions were used: 98°C for 3 min followed by 30 cycles of 98°C for 30 s, 55°C for 30 s, and 72°C for 2.5 min. The PCR was terminated by 5 min at 72°C. The resulting fragment was sub-cloned into the pJET2.1/blunt. For functional expression, FoxLOX was cloned into the pET28a-vector (yielding the pET28a/FoxLOX plasmid) and transformed into *E. coli* Bl21star cells (Invitrogen). Recombinant cells were cultivated at 37°C to OD_600_ of 0.6–0.8 either in LB-medium or 2xYT-broth. Expression was induced by the addition of 0.1 mM isopropyl β-D-thiogalactopyranoside. 0.1 mM ammonium iron citrate was added at the time point of induction in order to assure that LOX expression is not limited due to a low co-factor concentration. Cells were cultivated for 3 d at 16°C under constant shaking and harvested by centrifugation (8000×g, 20 min, 4°C). The resulting precipitate was frozen in liquid nitrogen and stored at −20°C.

### Protein Purification

The cell paste resulting from 1 L expression culture was thawed and resuspended in 50–100 mL lysis buffer: 50 mM Bis(2-hydroxyethyl)amino-tris(hydroxyl-methyl)methane pH 7.0 containing 20% (v/v) glycerol, 0.3% (w/v) 3-[(3-cholamidopropyl)dimethylammonio]-1-propanesulfonate, 0.5 M NaCl, 1 mM 1,4-dimercapto-2,3-butandiol and 0.2 mM phenylmethylsulfonylfluorid. Cells were lysed by the addition of 0.1 mg/mL lysozyme and incubation on ice for 30 min. After further disruption of the cells using a Sonifier® cell disruptor (B15, Branson), debris was removed by centrifugation at 50,000×g and 4°C for 20 min yielding cell free bacterial crude extract. The extract was either loaded on a pre-packed Protino® Ni-NTA Agarose (Macherey-Nagel) or on a His-Trap™ column (GE-Healthcare) that was pre-equilibrated with lysis buffer using an ÄKTAprime system (GE Healthcare). Proteins unspecifically bound to the column matrix were eluted from the column with at least 2–3 column volumes of lysis buffer containing 20 mM imidazol. FoxLOX was eluted using the same buffer containing 0.5 M imidazol. The purity of the protein was assessed via 10% SDS-PAGE and protein concentration was determined employing the method of Bradford [34]. Size exclusion chromatography was performed on an S200pg 26/60 column (GE-Healthcare) using 50 mM Tris(hydroxymethyl)amino-methane (Tris)/HCl-buffer pH 8.0 containing 10% glycerol and 125 mM NaCl.

### Analysis of the Metal Cofactor

The metal content of FoxLOX was analyzed by atomic emission spectroscopy employing an Optima 5500 DV (Perkin Elmer Precisely) in analogy to a method reported before [10]. The metal-cofactor of FoxLOX was removed by dialyzing the purified protein against 10 mM Tris/HCl-buffer, (pH 7.5), 2 mM ethylenediamine-tetraacetic acid, 380 µM desferrioxamine,0.5 M guanidinium chloride for 20 h at 4°C [35]. Reconstitution of the protein with iron or manganese was performed by incubating the apo enzyme with FeSO_4_ or MnCl_2_ (approx. 100 eq.), respectively, for 10 min. Unbound metal ions and residual chelator molecules were removed by dialysis against 50 mM Tris/HCl pH 7.5, 500 mM NaCl, 10% (v/v) glycerol.

### Determination of the pH-optimum

The pH-optimum was determined by measuring initial formation rates of the conjugated double bond system of the product at 234 nm (ε_234nm_[H(P)OD] = 2.3×10^4^ M^−1^cm^−1^) at a given pH-value in triplicates using a Cary 100 Bio spectrophotometer (Varian) as described elsewhere [10]. In order to prevent micelle formation, the sodium-salt of 18∶2(n-6) was used with a final concentration of 100 µM as substrate in the following buffer systems: 0.2 M acetate buffer (pH 4.0–5.5), 0.2 M phosphate buffer (pH 5.5–8.0), and 0.2 M borate buffer (pH 8.0–12).

### Analysis of Steady State Kinetics

Kinetic parameters were determined by measuring the time dependent initial formation rates of the conjugated double system of the product at 234 nm (ε_234nm_ for hydro(pero)xy octadecadi/trienoic acid) [H(P)OD/T] = 2.3×10^4^ M^−1^cm^−1^) using Cary 100 Bio spectrophotometer (Varian) in triplicates. The experiments were conducted at room temperature in 1 mL of 50 mM Tris/HCl buffer (pH 8.0) that was pre-equilibrated against air. The reaction was started by the addition of 10–100 µg protein and sodium salts of fatty acids were used as substrates. Kinetic parameters were determined by fitting 10–12 data points to the Michaelis-Menten equation using the Origin 8.5 software.

### Activity Assay and HPLC/DAD Product Analysis

To analyze the product specificity of FoxLOX either 10–100 µg purified protein (in 1 mL Tris/HCl pH 8.0) or cell free crude extract (1 mL) was incubated with 250 µg of 18∶2(n-6), 18∶3(n-3), 18∶3(n-6) or 20∶4(n-6) for 30–60 min at room temperature on a shaker. Incubations were stopped by the addition of 1 mL MeOH. In some experiments the MeOH contained 1–3 mg/mL SnCl_2_ that was used to reduce the hydroperoxy fatty acid products to the corresponding hydroxyl derivatives. Fatty acid derivatives were extracted with 1 mL CHCl_3._ For reversed phase (RP)-high performance liquid chromatography (HPLC) analysis the extracts were dried under continuous nitrogen stream, re-dissolved in acetonitrile/water/acetic acid (50/50/0.1, v/v/v), and further processed as described previously [36]. Briefly, fatty acids and the respective hydro(pero)xide derivatives were separated on a EX250/2 Nucleosil 120–5 C_18_ column (2.1×250 mm, 5 µm particle size; Macherey-Nagel). The following solvent system was used: acetonitrile/water/acetic acid (50/50/0.1, v/v/v) as solvent system A and acetonitrile/acetic acid (100/0.1, v/v) as solvent system B. The gradient elution profile employed in this study was: 0–5 min, 0.18 mL/min, 100% A; 5–20 min, 0.18 mL/min, 100% A to 100% B; 20–22 min, 0.18 mL/min to 0.36 mL/min, 100% B; 22–27 min, 0.36 mL/min, 100% B; 27–32 min, 0.36 mL/min, 100% B to 100% A; 32–35 min, 0.36 mL/min to 0.18 mL/min, 100% A. The analysis was performed on an 1100 HPLC system (Agilent) that was coupled to a diode array detector. Fatty acids where detected at 202 nm whereas hydroperoxide derivatives were detected at 234 nm (specific for conjugated diene system). ^14^C-labled metabolites were detected by a Raytest radio-detector. Separation of the positional hydroxyl isomers was performed using straight phase (SP)-HPLC. A Zorbax SIL column (2.1×150 mm, 5 µm particle size; Agilent) and a solvent system consisting of n-hexane/2-propanol/trifluoroacetic acid (100/1/0.02, v/v/v) was used for this analysis as described in [37]. The separation of stereo isomers via chiral phase (CP)-HPLC was performed on a Chiracel OD-H column (2.1×150 mm, 5 µm particle size; Baker) with a solvent system consisting of n-hexane/isopropanol/trifluoroacetic acid (100/5/0.1, v/v/v). Products were identified by comparison with authentic standards. Incubations of purified FoxLOX with 18∶2(n-6)-Me and di18∶2(n-6)-phosphatidylcholine (PC) were performed at room temperature in 1–5 mL 50 mM Tris/HCl (pH 8.0) containing 10% (v/v) glycerol, 300 mM NaCl, and 0.1% (v/v) sodium deoxycholate with 200–300 µg/mL of each substrate at room temperature for 60 min on a shaker. Control experiments were analogously performed with free 18∶2(n-6). The fatty acid hydroperoxides were reduced to their corresponding hydroxyl derivatives by the addition of SnCl_2_ (dissolved in MeOH as described above) and extracted with CHCl_3_. After extraction, the product(s) from incubations with free 18∶2(n-6) were methylated while those formed from di18∶2(n-6)-PC were transesterified as described above. Products were identified as described above by sequential RP-, SP-, CP-HPLC analysis as described above.

### HPLC/MS-analysis

HPLC/mass spectrometry (MS)-analysis was performed on a Surveyor HPLC system with an EC250/2 Nucleosil 100–5 C_18_-column (2.1×250 mm, 5 µm particle size; Macherey-Nagel) and the following solvent system according to [36]: acetonitrile/water/acetic acid (40/60/0.1, v/v/v) as solvent system A and acetonitrile/acetic acid (100/0.1, v/v) as solvent system B. The gradient elution profile was as follows: 0–10 min, 0.2 mL/min, 20% B; 10–30 min, 0.2 mL/min, 20% B to 100% B; 30–35 min 0.2 mL/min to 0.3 mL/min, 100% B; 35–40 min, 0.3 mL/min, 100% B; 40–44.5 min, 0.3 mL/min, 100% B to 20% B; 44.5–45 min, 0.3 mL/min to 0.2 mL/min 20% B. The mass spectrometer was a LCQ ion trap mass spectrometer (Thermo). The MS analysis was performed in the negative electron spray ionization mode scanning for negative ions. The capillary temperature was set to 300°C and the capillary voltage was 27 kV. Tandem-MS analysis was performed with collision energy of 1 V.

### Analysis of Products Formed from 18∶3(n-3)

In order to identify reaction products formed from 18∶3(n-3) as substrate additional experiments were performed with crude lysate that was obtained by sonification of bacterial cell pellets in 10 mL of sonication buffer (pH 7.4) and centrifugation at 48000×g. 2 mL of the resulting supernatant were diluted with 18 mL KPO_4_-buffer and incubated with 360 µM 18∶3(n-3) at 23°C for 30 min under stirring conditions. The material obtained after extraction with diethyl ether was treated with NaBH_4_ in MeOH in order to reduced hydroperoxides (and ketones) to hydroxides and subsequently methyl-esterified by treatment with diazomethane. An aliquot of this material was trimethylsilylated and analyzed by gas chromatography (GC)/MS using authentic oxylipins as references [38]. The remaining material was subjected to SP-HPLC [38] using a column of Nucleosil 50–5 (250×4.6 mm; purchased from Macherey-Nagel (Düren, Germany). Elution was performed with 0.7% isopropanol in hexane at 0–20 min with detection of products at 210 nm. More polar products were eluted subsequently with 5% isopropanol in hexane at 20–40 min with detection of products at 270 nm. Peaks were collected, trimethylsilylated and further analyzed by means of GC/MS before and after catalytic hydrogenation.

## Results

### Identification and Isolation of FoxLOX

**Figure 1:**
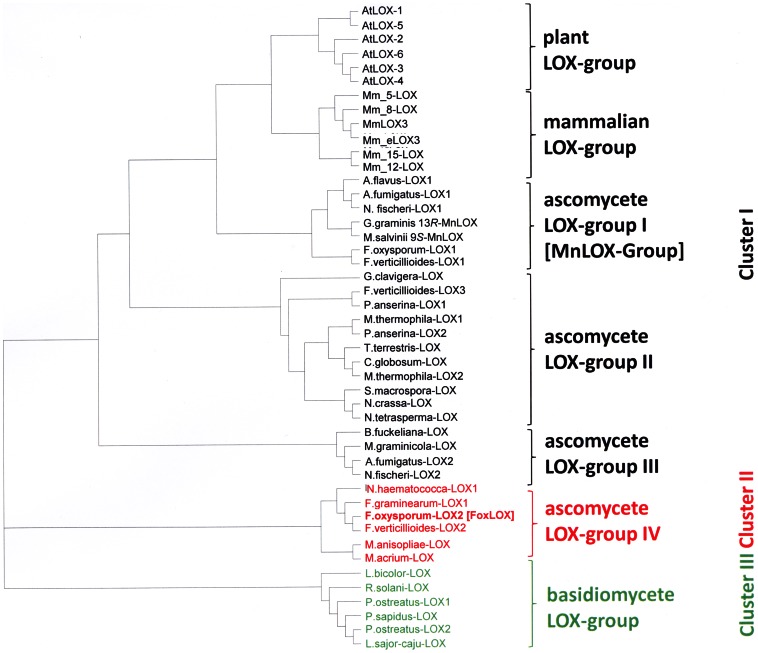
Phylogenetic analysis of FoxLOX. FoxLOX was analyzed with different LOXes from plants (*Arabidopsis thaliana*), mammals (*Mus musculus*) and mainly putative LOXes from different fungal species (*Aspergillus flavus, Aspergillus fumigatus, Botryotinia fuckeliana, Chaetomium globosum, Fusarium oxysporum*, *Fusarium graminearum*, *Fusarium verticillioides, Gaeumannomyces graminis, Grosmannia clavigera, Laccaria bicolor, Lentinus sajor-caju, Magnaporthe salvinii, Metarhizium anisopliae, Metarhizium acridum, Myceliophthora thermophila, Nectria haematococca, Neosartorya fischeri, Neurospora crassa*, *Neurospora tatrasperma, Pleurotus ostreatus, Pleurotus sapidus, Podospora anserina, Rhizoctonia solani, Sordaria macrospora, Thielavia terrestris*): *A. flavus*-LOX1 (AFL2G_06556.2); *A. fumigatus*-LOX1 (AFUA_4G02770); *A fumigatus*-LOX2 (Afu7g00860); *A. thliana*-LOX1 (At1g55020); *A. thaliana* LOX2 (At3g45140); *A. thaliana* LOX3 (At1g17420); *A. thaliana* LOX4 (At1g72520); *A. thaliana* LOX5 (At3g22400); *A. thaliana* LOX6 (At1g675609); *B. fuckeliana*-LOX (CCD50650.1); *C. globosum*-LOX (XP_001225066.1); *F. graminearum*-LOX1 (FGSG_02216); *F. verticillioides*-LOX1 (FVEG_05726.3); *F. oxysporum*-LOX1 (FOXG_02545.2); *F. oxysporum*-LOX2 [FoxLOX] (FOXG_04807); *F. verticillioides*-LOX3 (FVEG_09897.3); *F. verticillioides*-LOX2 (FVEG_03347.3); *G. graminis* 13*R*-MnLOX (AAK81882.1); *G. clavigera*-LOX (EFX02092.1); *L. bicolor*-LOX (XP_001881489.1); *L. sajor-caju*-LOX (CCV01579.1); *M. acridum*-LOX (EFY84539.1); *M. anisopliae*-LOX (EFZ04186.1); *M. graminicola*-LOX (EGP90986.1); *M. musculus* 5-LOX (NP_033792.1); *M. musculus* 8-LOX (NP_033791.1); *M. musculus* 15-LOX (NP_033790.3); *M. musculus* 12*R*-LOX3 (NP_035916.1); *M. musculus* 12*S*-LOX (P39655.4); *M. musculus* 12*R*-eLOX (O70582.1); *M. salvinii* 9*S*-MnLOX (CAD61974); *M. thermophila*-LOX1 (AEO59314.1); *M. thermophila*-LOX2 (AEO56683.1); *N. crassa*-LOX (CAD37061.1); *N. fischeri*-LOX1 (NFIA_030810); *N. fischeri*-LOX2 (NFIA_113540); *N. fischeri*-LOX3 (XP_001262720.1); *N. haematococca*-LOX1 (XP_003042428.1); *N. tetrasperma*-LOX (EGO61406.1); *P. anserina*-LOX1 (XP_003437217.1); *P.anserina*-LOX2 (XP_001911188.1); *P. ostreatus*-LOX1 (BAI99788.1); *P. ostreatus*-LOX2 (CCV01578.1); *P. sapidus-LOX* (CAQ87588.1); *R. solani*-LOX (CCO32261.1); *S. macrospora*-LOX (XP_003348064.1); *T. terrestris*-LOX (AEO63144.1). [35] The phylogenetic tree was constructed by using the ClustalX- software using default parameters. The tree was visualized using tree-view.

BLAST searches in the *F. oxysporum* genome (http://www.broadinstitute.org/annotation/genome/fusarium_group/MultiHome.html) using *Arabidopsis thaliana* LOX1 (At1g55020) and LOX2 (At3g45140) as templates for a 9-LOX and a 13-LOX, respectively, revealed two sequences with homology to a LOX. Further sequence analyses indicated that one of the sequences exhibited significant similarity to the previously described 13*R*-MnLOX from *Gaeumannomyces graminis* being specific for fungi. Analysis of the second sequence, however, suggested that it may encode a 13*S*-LOX similar to those found in the JA-biosynthetic pathway of plants. Therefore, only this sequence with a length of 2292 base pairs and 744 amino acids, respectively, was further analyzed. The open reading frame showed 26% similarity towards both template sequences and was termed FoxLOX (Broad Institute accession number FOXG_04807). A domain search using the NCBI database revealed the presence of a conserved LOX-type domain architecture for the C-terminal region (amino acids 243–622). However, for the N-terminal region no sequence motifs that are indicative for C2-domains, normally found in LOXes, could be identified. These domains are made up by a β-barrel-like structure and are discussed to regulate membrane binding and catalysis [39], [40]. For phylogenetic analysis putative LOX sequences of different fungal species (except 13*R*-MnLOX, here shown Gg_13*R*-MnLOX) as well as six LOX isoforms from *Mus musculus* (as representative of mammalian LOXes), and *A. thaliana* (as representative of plant LOXes) were used. As shown in Figure 1 related sequences are assembled in three major categories: Plant and mammalian LOXes, that each formed a sub-group by their own (termed: plant LOX group and mammalian LOX group), are clustered together with particular ascomycete LOXes that each form three different sub-groups and are assigned as ascomycete LOX group I-III. LOXes arranged in sub-group I are either shown (*G. graminis*_13*R*-MnLOX, *M. grisea*_9*S*-MnLOX) or predicted to contain manganese as catalytic metal instead of iron. The two further classes of related ascomycete LOX (ascomycete LOX-groups II and III) arranged in this cluster contain the majority of so far annotated but not further characterized fungal LOX-sequences. In contrast, the second major Cluster II notably contains only six different LOX sequences and also includes FoxLOX. LOX-sequences from basidiomycete organism are arranged in cluster III.

**Figure 2:**
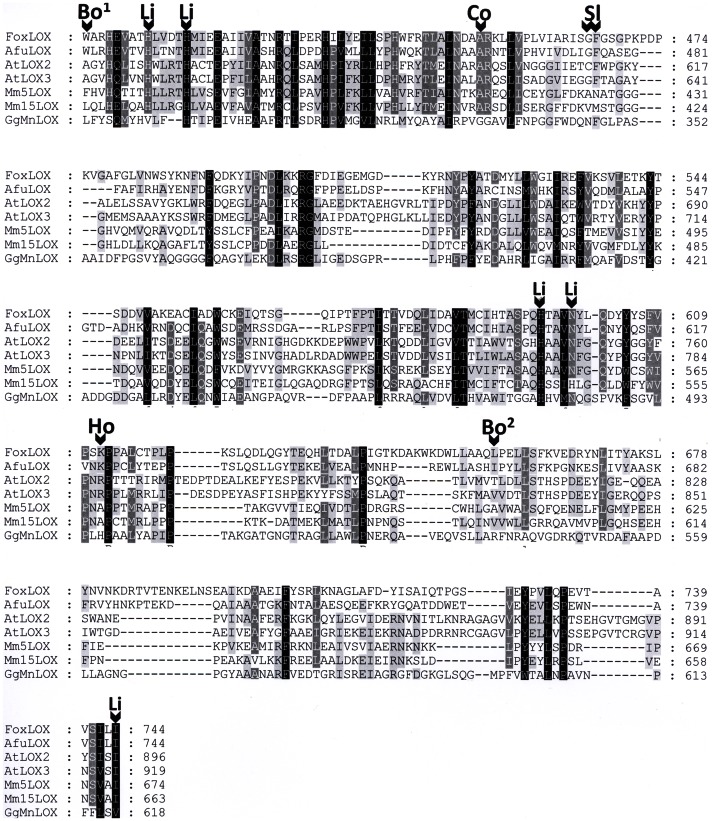
Partial amino acid alignment of different LOX forms with FoxLOX. Sequences were chosen from *F. oxysporum (Fox)*, *A. fumigatus (Af)*, *G. graminis (Gg)*, *A. thaliana (At)* and *M. musculus (Mm)*. Alignments were performed using the ClustalX software package employing default parameters. Different determinants for regio- and stereo specificity are indicated as “Bo” (Borngräber according to [66]), “Ho” (Hornung according to [44]), “Sl” (Sloane according to [43]) and “Co” (Coffa according to [45]). Amino acid residues involved in iron binding are indicated by “Li”.

Detailed sequence alignments were performed using amino acid sequences of two *A. thaliana* and two mouse LOXes as well as the *G. graminis* 13*R*-MnLOX (all in arranged in cluster I), the FoxLOX (cluster II), and one *A. fumigatus* LOX (cluster I, group III; Figure 2). This analysis showed that residues involved in coordinating the catalytic metal (either iron or manganese) are conserved in the FoxLOX sequence and are notably identical to that of plant LOXes as indicated in Figure 2 with the abbreviation “Li” (His-408, His-413, Asp-599, His-595, Ile-744). Several determinants responsible for the regio- and stereo specific dioxygenation reaction in mammalian and plant LOXes can also be identified in FoxLOX. In the following we will briefly highlight the underlying concepts explaining the molecular basis for regio- and stereo specificity and indicate the respective residues in FoxLOX: (i) In case of mammalian LOXes numerous studies established that regiospecificity is mainly determined by three amino acids forming the bottom of the active site and are thus determining the depth of substrate penetration (“triad concept”) [41]. The positions had been first described by Sloane (Sl) and Borngräber (Bo) [42], [43]. These three analogous positions are occupied by space filling amino acids in FoxLOX: Trp-400 (indicated as Bo^1^ in Figure 2), Phe-466 (indicated as Sl in Figure 2), and Leu-657 (indicated as Bo^2^ in Figure 2). (ii) In plants, on the other hand positional specificity has been suggested to be mainly determined by the orientation of the fatty acid within the active site [9], [37]. According to this model, 13-lipoxygenation occurs when the substrate enters the active pocket with its methyl end first. In analogy to the triad concept derived from mammalian LOXes, the penetration depth of this binding mode is determined by the same space filling residues described by Sloane and Borngräber [42], [43]. Then again, an inverse substrate orientation has been proposed for 9-lipoxygenation, according to which the substrate enters the active site with its carboxy-terminus first [37]. The negative charge of the carboxy terminus is then stabilized by a positively charged Arg residue, which in 13-LOXes is masked by bulky amino acids in the active site [44]. In case of FoxLOX this Arg is substituted by an also positively charged Lys (Lys-612 indicated as Ho) and a Phe residue (Phe-466 indicated as Sl) is positioned at the site of the “Sloane”-determinant which might shield the Lys as it has been observed in plant LOXes [44]. (iii) The stereo specificity of different LOXes is determined by a single Gly/Ala pair (“Coffa”-site) [45] that functions as a switch for “*R*”- and “*S*” selectivity [46]. This position is occupied in FoxLOX by an Ala-residue (Ala-451, indicated as Co). Considering all these different theoretical aspects, one may predict that FoxLOX catalyzes the “*S*”-specific oxidation of C_18_-polyunsaturated fatty acids at the C-13 and of C_20_-polyunsaturated fatty acids at the C-15.

For cloning of the FoxLOX gene, several methods have been initially employed in order to extract fungal RNA from the mycelium. In our experiments the TRIZOL-method gave the best and highest yield of RNA. Gene specific primers were used for the amplification from cDNA via PCR. Sequencing confirmed the ORF of 744 amino acids and no amino acid exchanges compared to the published sequence were discovered [47]. Optimal expression was achieved in *E.coli*-Bl21star cells at 16°C for 3 days.

### Purification and Oligomeric Structure

**Figure 3:**
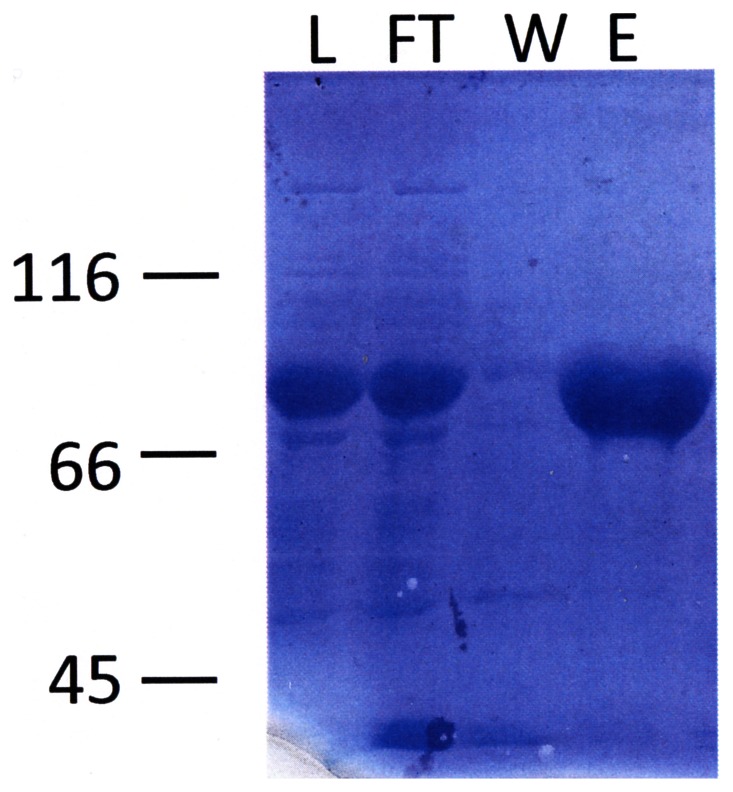
SDS-PAGE analysis of different fractions of FoxLOX purification. Shown is a 10% SDS-PAGE analysis of crude cell extract (load, L) applied on an immobilized Ni-column, unbound (flow through, FT), washed proteins (W) and eluted FoxLOX.

FoxLOX was expressed in-frame with an N-terminal hexa-histidin peptide that enabled the use of affinity chromatography as the major purification step (Figure S1A). This purification step resulted in a final purity of >95% as judged by SDS-PAGE analysis (Figure 3) and a yield of up to 30 mg protein/L of culture. Next the oligomeric state of FoxLOX was analyzed by size-exclusion chromatography. This analysis yielded a protein with a molecular mass of about 173 kDa (Figure S1B). This size is about twofold larger than that of the SDS-PAGE analysis (about 80 kDa) and the theoretical molecular weight of 83.5 kDa, suggesting that native FoxLOX may form a dimer in solution. While a similar oligomerisation has been reported for a few mammalian LOXes (e.g. human platelet 12-LOX [48]), plant LOXes (e.g. soybean LOX1 [49]) mainly form monomers in solution.

### Analysis of the Catalytic Metal

In order to analyze whether the metal bound in the active site of FoxLOX is an iron atom as for all plant and mammalian LOXes or a manganese atom as for 13*R*-MnLOX, the metal content of affinity purified enzyme was determined by atomic emission spectroscopy. In all preparations that were analyzed, only iron beside traces of manganese was detected, suggesting that iron is the catalytic metal in the active site of FoxLOX.

When the iron content was analyzed after size-exclusion chromatography, it was reduced by approx. 50% suggesting that it is only weakly coordinated by the enzyme. In order to test if the specific incorporation of the iron atom in the active site is caused by the heterologous expression system used or by the inherent protein architecture that precludes manganese from the active site, the iron atom was removed from the active site as reported before [35]. The enzyme was then reconstituted by dialysis in the presence of either manganese or iron, respectively. Atomic emission spectroscopy measurements of these enzyme preparations demonstrated that reconstitution in the presence of iron, but not in the presence of manganese led to incorporation of the metal atom. This finding thus confirmed that FoxLOX exhibits an active site architecture that specifically binds iron but not manganese.

### Analysis of the Fatty Acid Composition of *F. oxysporum*

In order to confirm endogenous substrates for FoxLOX catalysis, the composition of free as well as membrane bound fatty acids present in *F. oxysporum* mycelium were analyzed (Table 1). The results show that 18∶2(n-6), occurring free as well as in its esterified form, is the most abundant fatty acid. 18∶3(n-6) and 18∶3(n-3) were also detected as further potential FoxLOX-substrates but in lower amounts. Other fatty acids present in the mycelium were 16∶0, 16∶1(n-6), 18∶0, and 18∶1(n-9).

**Table 1:** Fatty acid composition of *F. oxysporum.*

Fatty acid	Free fatty acids Relative abundance [%]	Esterified fatty acids Relative abundance [%]
16∶0	32.1	15.2
16∶1(n-6)	1.3	2.3
18∶0	6.0	2.8
18∶1 (n-9)	9.8	16.2
18∶2 (n-6)	34.4	54.0
18∶3 (n-3)	5.5	9.1
18∶3 (n-6)	10.8	0.4

*F. oxysporum* was statically cultivated for 5 weeks in potato dextrose medium at 20°C. Fungal mycelium was grounded into a fine powder and extracted with CHCl_3_/MetOH and analyzed via GC/FID. Presented are mean values derived from two different experiments.

### pH-optimum

Initial experiments aimed to analyze the optimal pH-range for conversion of fatty acids by FoxLOX. For this propose FoxLOX was incubated with 100 µM 18∶2(n-6) in buffer systems with different pH-values and time dependent changes at 234 nm (formation of a conjugated diene system) were analyzed. While the catalytic activity of FoxLOX at acidic pH values (<pH 7.0) is considerably low, it increases rapidly at a neutral pH (pH 7.0) and reaches a maximum at pH 8.0. At higher pH, the activity drops to approx 75% of the maximal activity and remains constant up to pH 10.5. A more basic environment leads to a further decrease in catalytic activity (Figure S2).

### Product Specificity

**Figure 4:**
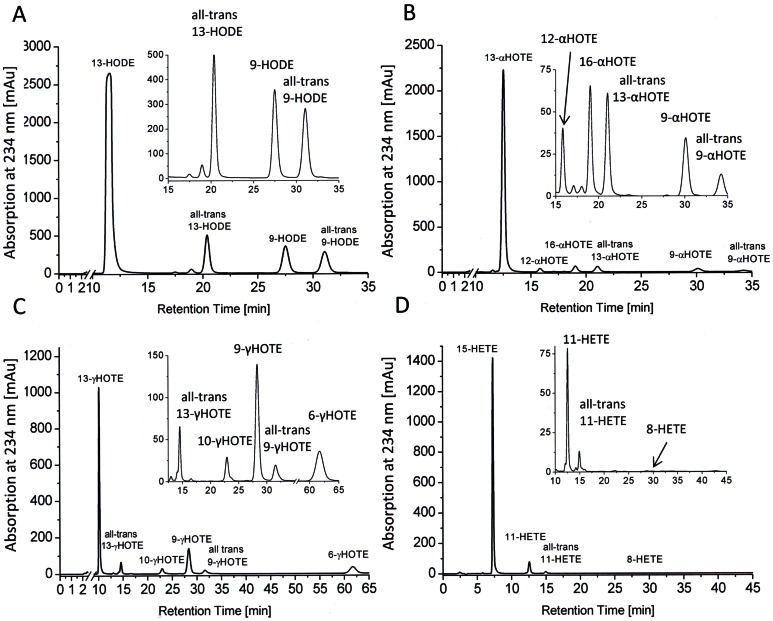
Product specificity of FoxLOX. SP-HPLC/DAD analysis at 234 nm of products formed from incubation of affinity purified FoxLOX with 18∶2(n-6) (A) 18∶3(n-3) (B) 18∶3(n-6) (C) 20∶4(n-6) (D) for 30–60 min and extractive isolation. Insets show an enlarged version of the respective region of the chromatogram. Before the analysis hydroperoxy fatty acids were reduced to the respective hydroxyl derivatives and pre-purified by RP-HPLC. Products were identified by comparison with authentic standards.

Next the oxygenation products formed from incubations with different C_18_ and C_20_-polyunsaturated fatty acids with FoxLOX were investigated. Chromatograms that were obtained from incubations of purified enzyme with 18∶2(n-6), 18∶3(n-3), 18∶3(n-6), and 20∶4(n-6) are shown in Figure 4. The quantitative analysis from incubations with crude cells lysates is summarized in Table 2. While all C_18_ fatty acids were predominantly converted to the 13-hydro(pero)xide derivatives, 20∶4(n-6) was mainly converted to the 15-hydro(perox)y-derivative. CP-HPLC analysis revealed that the main products were formed specifically in the *S*-configuration.

**Table 2:** Products formed from incubations of different fatty acid substrates and crude cell extracts of FoxLOX-expressing *E. coli* Bl21star cells.

Substrate	Products	Percentage (%)	*S* enantiomer (%)
	13−/9−/	74±3/9±1/	100/30/
18∶2(n-6)	all-trans13−/all-trans 9-	9±1/8±1	80/99
	Hydro(pero)xy octadecadienoic acid		
	16−/13−/12−/9−/	5±2/83±2/5±4/4±1/	60/100/63/55/
18∶3(n-3)	all-trans 13−/all-trans 9-	2±2/1±1	93/85
	Hydro(pero)xy octadecatrienoic acid		
	13−/10−/9−/6−/	52±1/5±1/22±0/14±1/	80/59/55/57/
18∶3(n-6)	all-trans 13−/all-trans 9-	6±1/4/±1	65/92
	Hydro(pero)xy octadecatrienoic acid		
20∶4(n-6)	15−/11−/8-	92±15/8±4/0±1	96/75/n.a.
	Hydro(pero)xy eicosatetraenoic acid		

After incubation for 30–60 min and extractive isolation the hydroperoxy fatty acids were reduced to the respective hydroxyl derivatives. The analysis was performed using SP-HPLC/DAD and CP-HPLC/DAD, respectively, at 234 nm. For quantification the peak areas were integrated. Products were identified by comparison with authentic standards. The results are presented as mean values ± standard deviation derived from at least three different experiments.

Notably, incubations with 18∶3(n-3) yielded four additional products. When the conversion of 18∶3(n-3) was photometrically monitored the absorption at 234 nm rapidly increased due to the formation of hydroperoxide products. In parallel the absorption around 280 nm increased continuously. After 2 min, however, the absorption at 234 nm started slowly to decrease while the absorption around 280 nm further increased giving an isosbestic point at approx. 250 nm (Figure S3A) suggesting that the hydroperoxy product was further metabolized. As shown in Figure S3B similar incubations with 18∶2(n-6) only led to a time dependent increase in the absorption at 234 nm. Since no other spectral changes were observed, this finding suggests that the further metabolization of the hydroperoxy product by FoxLOX is restricted to 18∶3(n-3) as substrate. This was further supported by experiments in which the product profile from incubations of FoxLOX with ^14^C-labeled 18∶2(n-6) and 18∶3(n-3) were analyzed as shown in Figure S4. While 18∶2(n-6) was mainly oxidized yielding the respective hydro(pero)xy derivative, 18∶3(n-3) was converted to a set of products having different spectral properties. In order to analyze those metabolites, the products formed by incubations with non-labeled 18∶3(n-3) were analyzed by RP-HPLC/DAD, RP-HPLC/MS^2^, SP-HPLC, and GC/MS.

**Figure 5:**
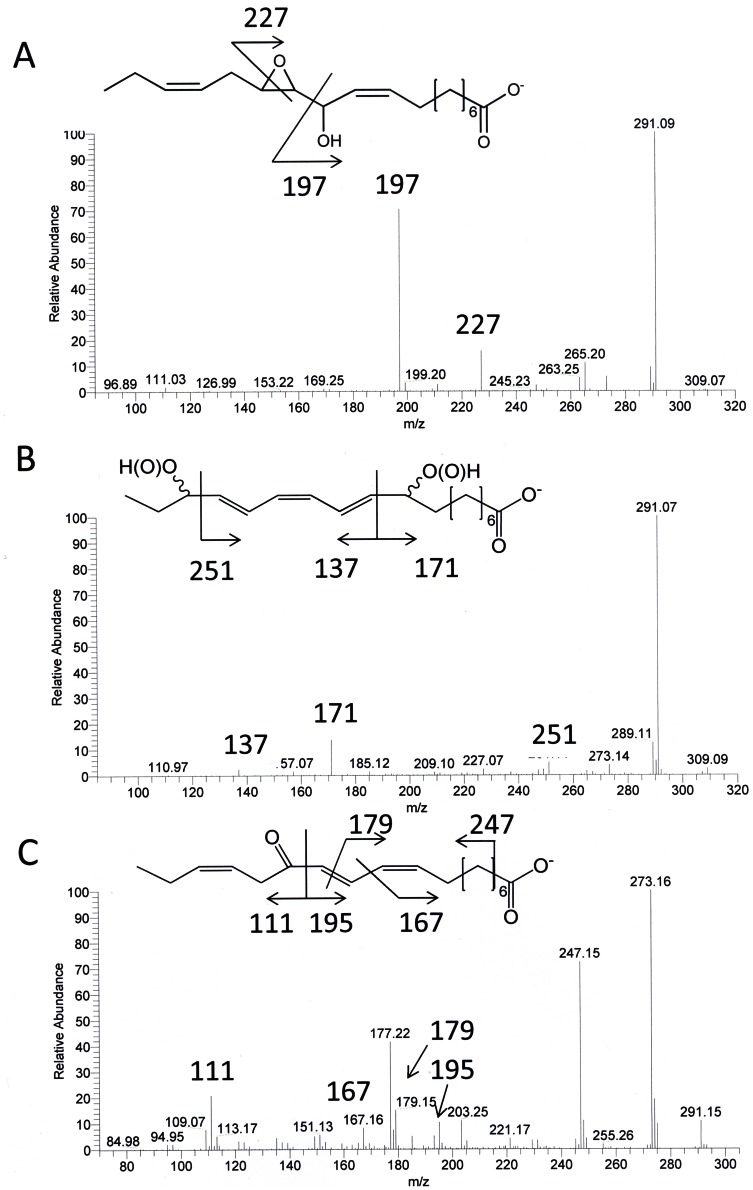
MS analysis of products formed from 18∶3(n-3) and fatty acid hydroperoxidase activity of FoxLOX. Shown are the MS^2^ spectra of 12,13-Ep-11-HOD (A), 9,16-DiH(P)OT (B) and 13-KOT derived from incubations of 18∶3(n-3) and FoxLOX.

The absorption properties of two of these products were characteristic for a system with three conjugated double bonds giving maxima at 260, 270, and 280 nm (Figure S4A). The MS^2^ spectra of both products were highly similar and showed the following fragments being characteristic for 9,16-dihydroxy octadecatrienoic acid (DiHOT) [50] (Figure 5B): *m/z* 291 (M^−^–18; loss of water), *m/z* 251 (cleavage between C-15 and C-16), *m/z* 171/137 (cleavage between C-10 and C-11). On SP-HPLC this compound also gave two peaks with a 1∶1-ratio and subsequent GC/MS showed identical fragmentation spectra for both peaks that were also characteristic for 9,16-DiHOT. Based on these findings, these products were assigned as two diastereoisomers of 9,16-DiHOT.

In further experiments, reduced and methyl-esterified products generated from 18∶3(n-3) were analyzed by a combination of SP-HPLC/DAD and GC/MS. As seen in Figure S5, the HPLC chromatogram displayed several peaks of material absorbing at 210 and/or 270 nm. Materials giving rise to peaks 1–7 were collected, trimethylsilylated, and analyzed by GC/MS before and after catalytic hydrogenation. In this way, the following compounds could be identified: 13(*S*)-HOT (peak 1), 12*S*,13*S*-epoxy-11-hydroxy-9*Z*,15*Z*-octadecadienoic acid (peak 2; ratio between *threo* and *erythro* forms, 93∶7), 9-methoxy-16-hydroxy-10,12,14-octadecatrienoic acid (two isomers in 1∶1 ratio, peaks 3 and 4), 15,16-dihydroxy-9,11,13-octadecatrienoic acid (peak 5; ratio between *erythro* and *threo* forms, 81∶19), and 9,16-dihydroxy-10,12,14-octadecatrienoic acid (two isomers in 1∶1 ratio, peaks 6 and 7).

Additional support for the epoxy alcohol structure, *i.e.* 12,13-epoxy-11-hydroxy octadecadienoic acid, was provided by the mass spectrum of the non-derivatized compound which showed *m/z* 291 (M^−^–18; loss of water), *m/z* 265 (M^−^–44; loss of CO_2_), *m/z* 227 (cleavage of the oxirane ring), *m/z* 197 (cleavage between C-11 and C-12) (Fig. 5A). As a further product 13-keto octadecatrienoic acid (13-KOT) was detected. This compound had an absorption maximum at 279 nm and showed a characteristic MS^2^ spectrum [18] (Figure 5C) with the fragmentation pattern: *m/z* 273 (M^−^–18; loss of water), *m/z* 247 (M^−^–44; loss of CO_2_), *m/z* 195/111 (cleavage between C-12 and C-13), *m/z* 177 (295- 18; loss of water) and, *m/z* 167 (cleavage between C-10 and C-11). GC/MS-analysis also confirmed the formation of 13-KOT.

### Kinetic Parameters

Kinetic properties of FoxLOX were analyzed by incubating the purified enzyme with different substrates at pH 8.0. The initial rate of product formation at 234 nm was spectroscopically measured, derived data points were fitted to the Michaelis-Menten equation (shown for 18∶2(n-6) in Figure S6), and determined k_cat_- and K_M_-values are summarized in Table 3. Based on the k_cat_/K_M_-values these results show that FoxLOX can oxidize C_18_ and C_20_ fatty acids to similar extend. It should be noted, however, that compared to the other fatty acid substrates the k_cat_/K_M_-value obtained for 18∶3(n-3) was highly reduced: While the K_M_-value for this substrate was very similar (K_M_ = 31.7 µM) to the one for 18∶3(n-6) (K_M_ = 34.8 µM), the k_cat_-values for both substrates differed significantly by the factor of about 10 (k_cat_ [18∶3(n-3)] = 93.93±3.21 min^−1^ *vs.* k_cat_ [18∶3(n-6)] = 1114.64±102.76 min^−1^). Considering that hydroperoxy products formed from 18∶3(n-3) are further metabolized into above mentioned epoxy alcohol-, dihydroperoxy-, and keto fatty acids, all having absorption properties that are remarkably different from that of HPOTs, this fact might explain the molecular basis for the apparent different kinetic parameters.

**Table 3:** Kinetic properties of FoxLOX.

Substrate	K_M_ [µM]	K_cat_ [min^−1^]	K_cat_/K_M_ [min^−1^M^−1^×10^6^]
18∶2(n-6)	6.89±0.79	155.66±7.22	22.59
18∶3(n-3)	31.69±3.79	93.93±3.21	2.96
18∶3(n-6)	34.84±5.38	1114.64±102.76	31.99
20∶4(n-6)	3.37±0.55	102.57±3.79	30.44

Kinetic constants were spectrophotometrically analyzed by determining the initial rate of product formation at 234nm at different substrate concentrations. All experiments were performed in 50 mM Tris/HCl pH 8.0 at room temperature using a Varian Cary 300. The results are presented as mean values ± standard deviation derived from triplicate measurements.

### Analysis of the Dioxygenation Mechanism

**Figure 6:**
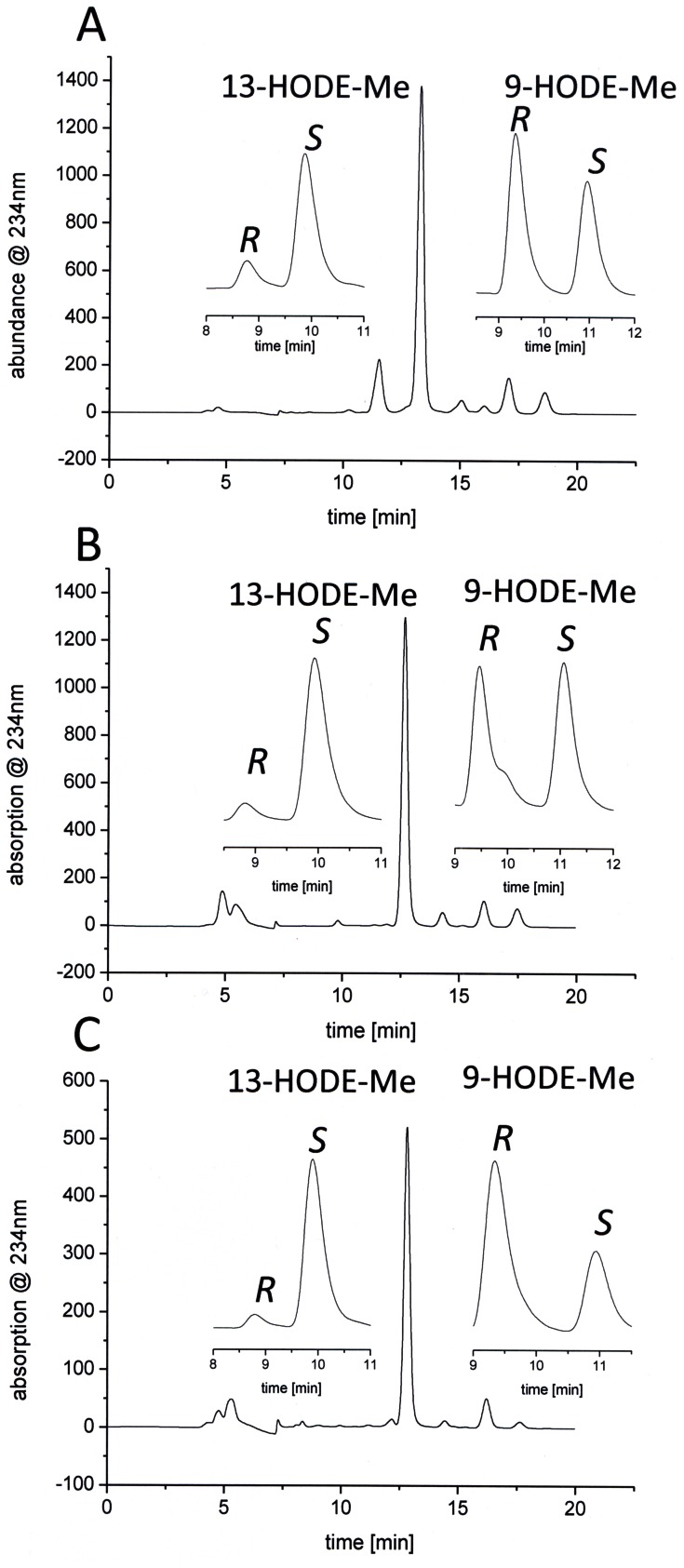
HPLC analysis of the products formed from 18∶2(n-6) (A), 18∶2(n-6)-Me (B) and di18∶2(n-6) PC (C). Reactions were performed in 50 mM Tris/HCl (pH 7.5) containing 10% glycerol, 0.1% sodium desoxycolate and 300 mM NaCl at room temperature for 60 min. Incubations with 18∶2(n-6) and di18∶2(n-6) PC were methylated or transmethylated, respectively. Before extraction the hydroperoxy fatty acids were reduced to their corresponding hydroxyl derivatives. The analysis was carried out by using SP-HPLC. As insets the CP-HPLC of each product is shown.

In recent studies fatty acid esterified to PC has been used to analyze the orientation of substrate during catalysis [10], [17]. In a similar approach di-18∶2(n-6)-PC was used here. As control experiments similar incubations were performed with 18∶2(n-6) and its methyl ester (18∶2(n-6)-Me). The resulting products were analyzed as the respective methyl ester derivatives. As shown in Figure 6 di-18∶2(n-6)-PC as well as free 18∶2(n-6), and its methyl ester were specifically converted to 13*S*-hydroperoxy octadecadienoic acid (13*S*-H(P)OD) as the main reaction product indicating that a tail-first binding takes place prior to the enzymatic conversion of the substrate.

**Figure 7:**
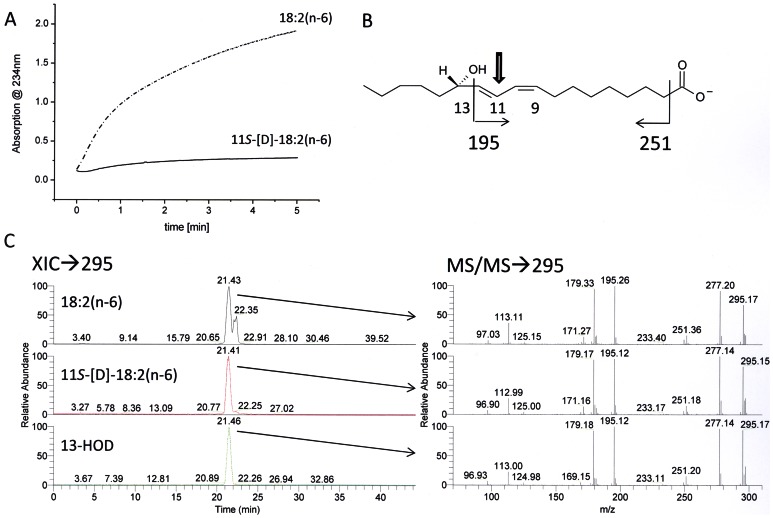
Analysis of the oxygenation mechanism using stereo specifically deuterated 11*S*-D-18∶2(n-6). (A) Shown is a progress curve at 234nm of the incubation of 100 µM 18∶2(n-6) and 11*S*-D-18∶2 (n-6), respectively, and 5 µg affinity purified FoxLOX in 200 µL 50 mM Tris/HCl (pH 8.0). After incubation for additional 30 min the products were extracted and analyzed by using RP-HPLC/MS^2^. (B) Shown is a structure of 13-HOD (carboxylate anion) with the formation of major fragment ions and (C) an extracted ion chromatogram (XIC) of a mass range of 294.5–295.5 amu with the respective tandem-MS spectra.

The stereochemistry of the hydrogen removal taking place from C-11 of the fatty acid backbone was next determined. For this purpose, the enzyme was incubated with stereospecifically labeled [11*S*-^2^H]18∶2(n-6) and kinetic parameters were determined. As shown in Figure 7A, conversion of this deuterated substrate took place at a significantly lower rate compared to that observed with unlabeled 18∶2(n-6), corresponding to a kinetic isotope effect (KIE) of approx. 15. This finding is in agreement with data observed for plant and mammalian LOXes for which unusually large KIEs have been reported in similar experiments [51], [52] and thus suggests that the 11*S*-hydrogen is selectively abstracted during catalysis. Indeed, when the products formed from labeled and unlabeled substrate were analyzed, no differences in their respective MS^2^ spectra were observed, confirming that the product did not contain any deuterium label (Figure 7C). Since the hydroperoxide formed has mainly the *S* configuration, these results demonstrate that the oxygenation reaction proceeds via an antarafacial mechanism as it is known from all iron containing LOXes so far.

## Discussion

Previous studies demonstrated that the plant pathogen *F. oxysporum* is capable of synthesizing the hormone JA [24] which controls plant development as well as stress responses [28]. The fact that a fungus is able to produce plant-specific signaling molecules led to the concept that these metabolites may interfere with those of the host in order to manipulate its defense reactions [4], [53]. However, in contrast to the well studied JA biosynthetic pathway in plants, that operating in fungi is still elusive. A recent study on this topic demonstrated that in the fungus *L. theobromae* JA is derived from a fatty acid precursor and is formed via the intermediary formation of a cyclopentenone suggesting that the fungal pathway might be similar to that of plants [26]. On the other hand a subsequent report, which aimed to characterize the dioxygenase putatively involved in this biosynthetic pathway, could not confirm the existence of plant-like JA biosynthetic enzymes in *L. theobromae.* Instead, an unusual 9*R*-dioxygenase that catalyzing the suprafacial oxidation of C_18_-fatty acids to the corresponding 9*R*-hydroperoxy fatty acids was detected [30]. The aim of the present study was to identify lipoxygenase activity needed for biosynthesis of JA in *F. oxysporum* by using a reversed genetic approach based on the genome of this fungus. Of the two predicted LOXes from *F. oxysporum*, the one encoded for by the gene FOXG_04807.2 was chosen. The enzyme "FoxLOX" was cloned from fungal cDNA, heterologously expressed in *E. coli,* and purified by affinity chromatography. To the best of our knowledge FoxLOX is the first ascomycete non-heme iron containing LOX that has been cloned and recombinantly expressed in *E. coli* and enzymatically characterized.

Phylogenetic analysis shows that at least four major clades of LOX may exist in ascomycetes; all being evolutionary distinct from previously characterized LOXes from plants and mammals (Figure 1). While the mammalian and plant LOXes were grouped in one major cluster together with the 13*R*-MnLOX, FoxLOX interestingly grouped in a second comparatively small cluster of ascomycete LOXes suggesting that there may be remarkable differences not only in the amino acid sequence, but in their enzymatic properties. These differences are on the one hand reflected by the observation that only the C-terminal catalytic domain of FoxLOX is similar to all other known eukaryotic LOXes. No sequence motif that is indicative for a C2-domain, normally found in the N-terminus of LOXes, could be identified in the N-terminal region of FoxLOX. On the other hand the C-terminal region of FoxLOX was found to harbor specific amino acid positions, known to determine the regio- and stereo specific dioxygenation of fatty acids by mammalian and plant LOXes [9] (Figure 2). Based on this analysis, we predicted FoxLOX to be a linoleate 13*S*- and an arachidonate 15*S*-LOX and this could indeed experimentally be confirmed (Table 2 and Figure 4). Hence, these results suggest that the concepts which were established over the last years for plant [9] and mammalian LOXes [54] in order to explain the molecular basis for regio- and stereo specific dioxygenation can also be applied to FoxLOX. Another feature that points to a similarity of FoxLOX to classical LOXes from plants and mammals is the presence of iron in the active site. This finding is interesting since the only ascomycete LOX that has been cloned and recombinantly expressed so far besides FoxLOX, is the manganese-dependent 13*R*-MnLOX from *G. graminis* [55].

Two more features of FoxLOX support the observation that this enzyme is more related to plant and mammalian LOXes than to the fungal 13*R*-MnLOX: (i) It catalyzes a classical antarafacial hydrogen abstraction and oxygen insertion as it has been found for all iron LOXes described so far (Figure 7). (ii) Its reaction has a KIE of approx. 15. This value is higher than the semi-classical limit and thus suggests that the hydrogen abstraction occurs as rate limiting step by quantum mechanical tunneling [56]. It should be mentioned, however, that compared to the much higher values reported for other LOX-forms (KIE ∼ 25≤80) [57], [58], the value of 15 may suggest a slightly different catalytic mechanism, but we did not follow up a detailed investigation of the underlying biophysical factors causing this differences.

**Figure 8:**
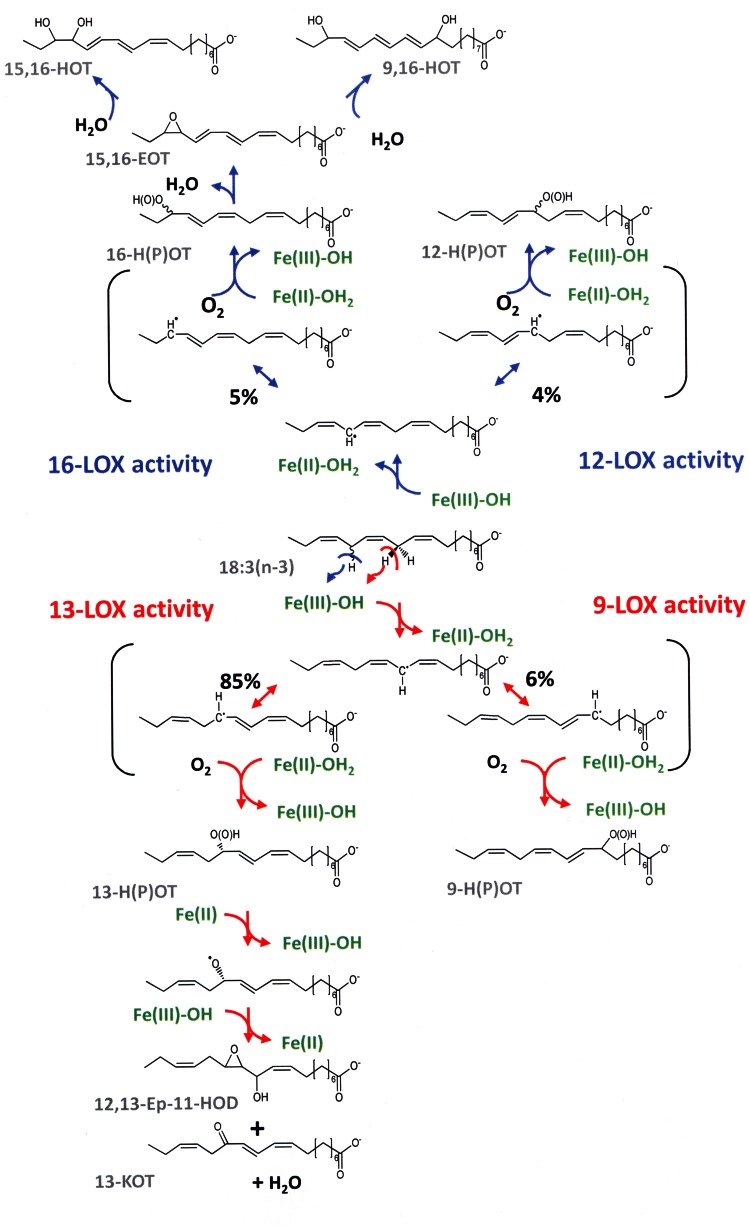
Conversion of 18∶3(n-3) by FoxLOX leading to a set of different products. The reaction starts with stereo specific abstraction of a specific hydrogen either from the C-11 or C-14 of the fatty acid backbone yielding a carbon centered radical that is delocalized via the π-electron system. In case of the predominant 13-LOX activity the pro*R*-hydrogen is abstracted from the C-11 and oxygen is inserted antarafacially at the C-13 yielding 13*S*-HPOT. The product is further converted either by isomerization yielding 12,13-Ep-11-HOD or dehydration forming 13-KOT. In case of 9-LOX activity 9-HPOT is formed by hydrogen insertion at the C-9. Abstraction of the hydrogen at the C-14 leads analogously to the formation of a delocalized carbon centered radical that can be either trapped at the C-12 (12-LOX activity) or at the C-16 (16-LOX activity) yielding 12-H(P)OTE or 16-H(P)OTE, respectively. The latter product might be further converted to 15,16-EpOT by elimination of a hydrogen from the C-11. The epoxy-group of this leukotriene A like metabolite can be attacked by water either at the C-15 (yielding 15,16-DiHOT) or at the C-9 (yielding 9,16-DiHOT).

Interestingly, in contrast to other fatty acids incubations of 18∶3(n-3) with FoxLOX at high substrate concentrations not only led to the formation of a hydroperoxy fatty acid product, but also yielded at least six different additional products which were derived from the further conversion of the hydroperoxy fatty acid (Figure 5). Among those we identified the epoxy-alcohol *threo*-12,13-epoxy-11-hydroxy-9,15-octadecadienoic acid and the keto-derivative 13-KOT. The finding that these products were only formed in minor amounts when using moderate 18∶3(n-3) concentrations might indicate that even under stirring conditions the reaction was easily depleted of dioxygen. Besides the above mentioned products we also observed formation of doubly oxidized fatty acid, i.e. 9,16-DiHOT and 15,16-DiHOT. 9,16-DiH(P)OD has been isolated as products of potato tuber LOX [59] and also by two enzymes from cyanobacteria - CspLOX2 from *Cyanothece spec* [10] and AOS-LOX fusion from *Nostoc punctiforme* [50]. For the latter enzyme substrate labeling as well as enzyme-truncation studies suggested that the dihydroxy fatty acid was derived from the 9-hydroperoxy fatty acid by the catalytic activity of the N-terminal AOS domain and that the second hydroxyl group originates from water rather than from molecular oxygen [50]. Formation of 15,16-DiHOT together with a 1∶1 ratio of 9,16-DiHOT diastereomers in incubations of 18∶3(n-3) with FoxLOX suggested a leukotriene-like mechanism involving intermediate formation of 15,16-epoxy-9,11,13-octadecatrienoic acid (Fig. 8). It is interesting to note that this epoxytriene has to be much more stable than *e.g.* leukotriene A_4_, since it survived extraction with diethyl ether and was opened into the two diastereomeric 9-methoxy-16-hydroxyoctadecatrienes (Figure S5) only when stored for longer periods in methanol.

Besides the dihydroxy-products varying amounts of 13-KOT and the epoxy-alcohol 12,13-Ep-11-HOD were also detected. They were formed by dehydration and rearrangement of the respective 13-hydroperoxy product. The fact that this reaction was observed under conditions where no metal-reducing agents were present indicates that this activity is distinct from the pseudo-peroxidase activity reported for soybean LOX1 [60] and mammalian 5-LOX [61]. In fact the inherent isomerase activity resembles those reported for a mutant of 13*R*-MnLOX from *G. graminis* and eLOX3. The latter enzyme is involved in the formation of skin barrier lipids [62] and is unique in the fact that it exhibits predominant hydroperoxide isomerase activity. Only recently the dioxygenase activity of this enzyme was unveiled by using synthetic fatty acid substrates and appropriate enzyme activators [63]. A related multifunctional activity of plant LOXes has only been reported for a LOX from the moss *Physcomitrella patens* [64], [65]. In 13*R*-MnLOX production of epoxy alcohols was only observed for a variant in which the Gly/Ala-pair at the so-called Coffa-site was mutated [18]. Together with neighboring Leu- and Phe residues this Gly may form a hydrophobic substrate channel which may be in close proximity to the catalytic metal. The importance of these amino acids on catalysis has been studied recently by site directed mutagenesis and it was shown that this region is not only determining the positional but also the stereo specificity of 13*R*-MnLOX and is thus forming a switch for regulation of suprafacial *vs.* antarafacial oxidation [20].

In conclusion, we cloned and characterized the first fungal non-heme iron containing linoleate 13*S*-LOX. Although the enzyme exhibits similar mechanistic properties to classical iron LOXes from plants and mammals (antarafacial reaction mechanism, tail-first substrate binding and large KIE), it exhibits with 18∶3(n-3) as substrate additional hydroperoxidase activity which converts the product hydroperoxides into dihydroxy-, keto-, and epoxy alcohol derivatives. The identification of FoxLOX as a specific linoleate 13*S*-LOX might hint towards a JA biosynthetic pathway in *F. oxysporum* which is analogous to that in plants.

## Supporting Information

Figure S1 **Purification of FoxLOX via affinity (A) and size exclusion chromatography (B).**
(TIF)

Figure S2 **Analysis of the pH-Optimum of FoxLOX activity.** Initial time dependent changes at 234 nm were determined in buffer systems with different pH-values (0.2 M acetate buffer (pH 4.0–5.5), 0.2 M phosphate buffer (pH 5.5–8.0), and 0.2 M borate buffer (pH 8.0–12).The sodium-salt of 18∶2(n-6) was used with a final concentration of 100 µM as substrate.
(TIF)

Figure S3 **Analysis of time dependent changes in the absorption properties of products formed from incubations of FoxLOX with (A) 18∶3(n-3) and (B) 18∶2(n-6), respectively.** Product spectra were recorded for 16 min at different time points using the scanning kinetic technique.
(TIF)

Figure S4 **Analysis of the product profile formed from incubations of (A) [1-^14^C]-18∶3(n-3) and (B) [1-^14^C]-18∶2(n-6) with FoxLOX.** A representative radio-HPLC product profile that was derived from incubations of FoxLOX with [1-^14^C]-18∶3(n-3) and [1-^14^C]-18∶2(n-6) is shown in (A) and (B), respectively. The analysis was carried out on a RP-HPLC-system that was coupled to diode-array- and a radio-detector. While chromatogram displays the signals recorded by the radio-detector, the insets show the absorption spectra of the respective products that were record by the diode-array detector. It should be emphasized that the products, shown in this figure, were only assigned based on their retention time, spectral properties and in accordance to data from different other independent experiments (*cf.* figure S5). Please also note that due to different levels of DiHOT-derivatives detected in this experiment, apparent differences in the respective absorption properties are caused. Therefore the DiHOT-2 product was only tentatively assigned, because its high amounts may led to absorption signals reaching the saturation of the diode array detector leading suboptimal spectral resolution.
(TIF)

Figure S5 **Separation of reduced and methyl-esterified products of 18∶3(n-3) oxygenation by FoxLOX.** Elution was accomplished at a rate of 2 mL/min with 0.7% 2-propanol/hexane (0–20 min; UV detection at 210 nm) followed by 5% 2-propanol/hexane (20–40 min; UV detection at 270 nm). Materials forming peaks 1–7 were collected and identified by GC-MS as follows: 13-HOT methyl ester (peak 1), methyl 12*S*,13*S*-11-hydroxy-9(*Z*),15(*Z*)-octadecadienoate (peak 2; ratio *threo*/*erythro* forms, 93∶7), methyl 9-methoxy-16-hydroxy-10,12,14-octadecatrienoate (diastereomers in 1∶1 ratio; peaks 3 and 4), methyl 15,16-dihydroxy-9,11,13-octadecatrienoate (peak 5; ratio *erythro*/*threo* forms, 81∶19), and methyl 9,16-dihydroxy-10,12,14-octadecatrienoate (diastereomers in 1∶1 ratio; peaks 6 and 7).
(TIF)

Figure S6 **Analysis of steady state kinetics from incubations of FoxLOX with different concentrations of 18∶2(n-6).** Initial time dependent changes at 234 nm were determined at different substrate concentrations. The results are presented as mean values ± standard deviation derived from triplicate measurements. Data points were plotted and fitted by employing the Michaelis-Menten equation to derive the kinetic constants.
(TIF)
