# Supplementary figures and images for: An Iron 13S-Lipoxygenase with an α-Linolenic Acid Specific Hydroperoxidase Activity from Fusarium oxysporum
Source: PLoS One. 2013 May 31;8(5):e64919. doi: 10.1371/journal.pone.0064919 (PMC3669278; doi:10.1371/journal.pone.0064919)

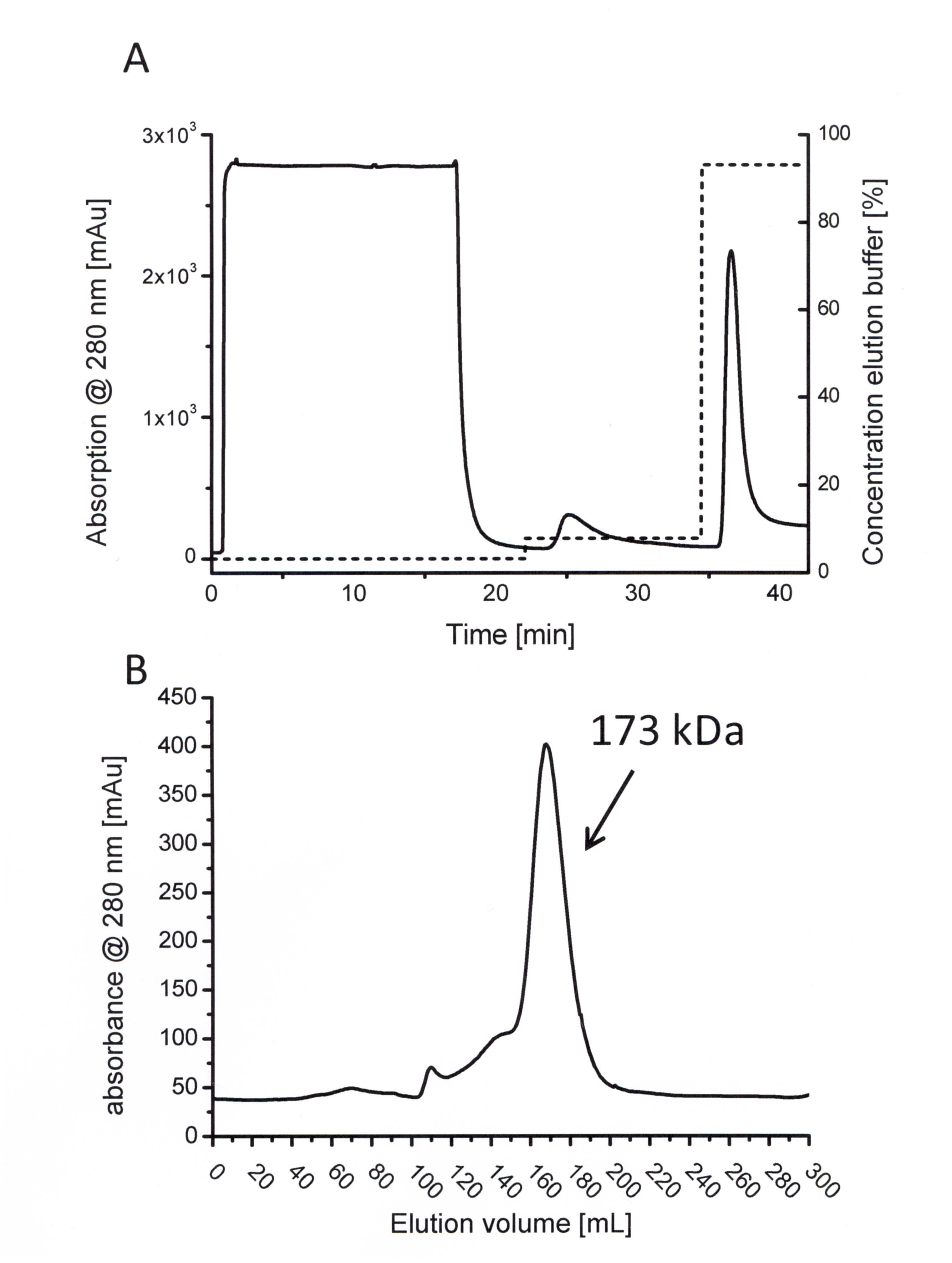

Supplement: Figure S1 — Purification of FoxLOX via affinity (A) and size exclusion chromatography (B). (TIF) [file pone.0064919.s001.tif]

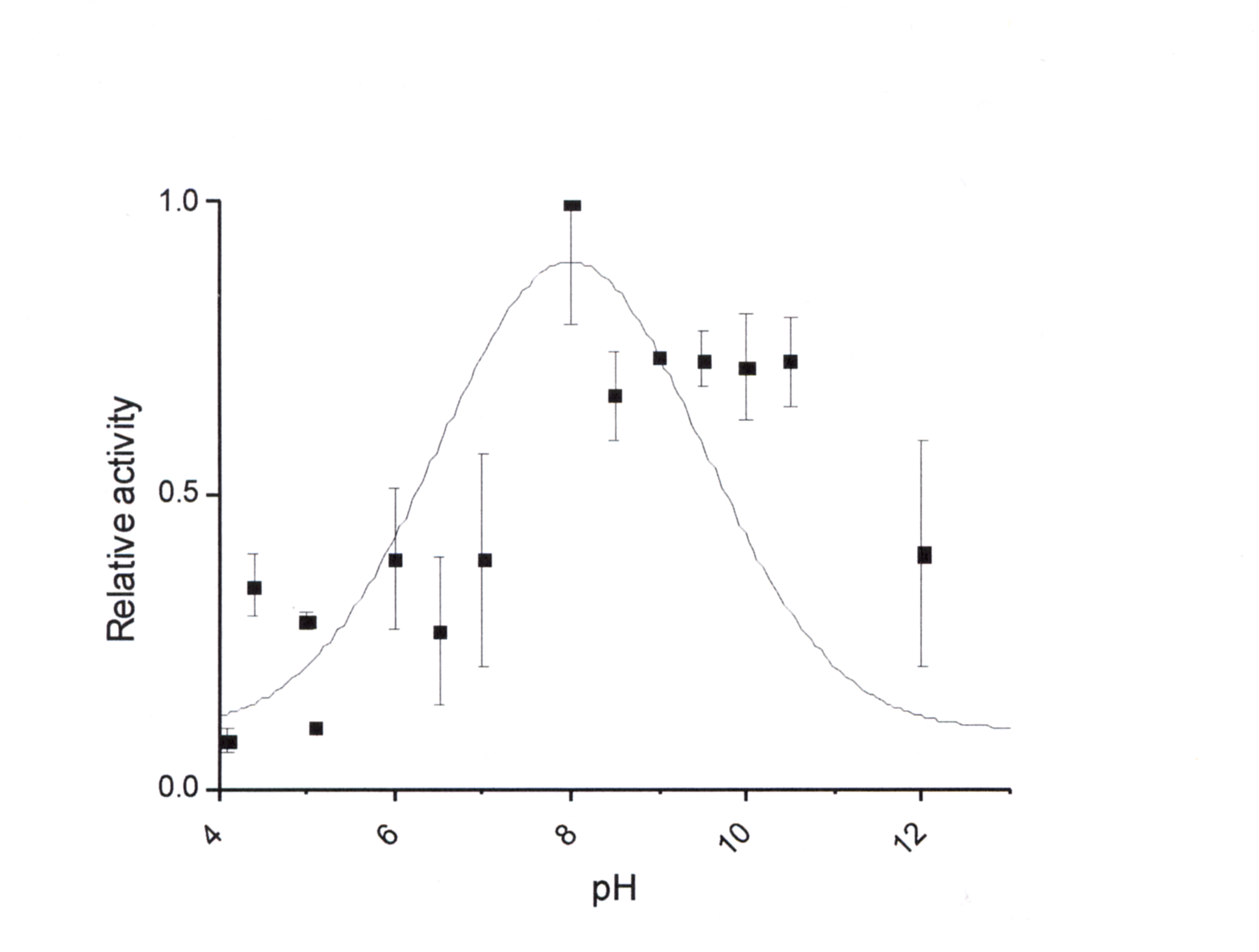

Supplement: Figure S2 — Analysis of the pH-Optimum of FoxLOX activity. Initial time dependent changes at 234 nm were determined in buffer systems with different pH-values (0.2 M acetate buffer (pH 4.0–5.5), 0.2 M phosphate buffer (pH 5.5–8.0), and 0.2 M borate buffer (pH 8.0–12).The sodium-salt of 18∶2(n-6) was used with a final concentration of 100 µM as substrate. (TIF) [file pone.0064919.s002.tif]

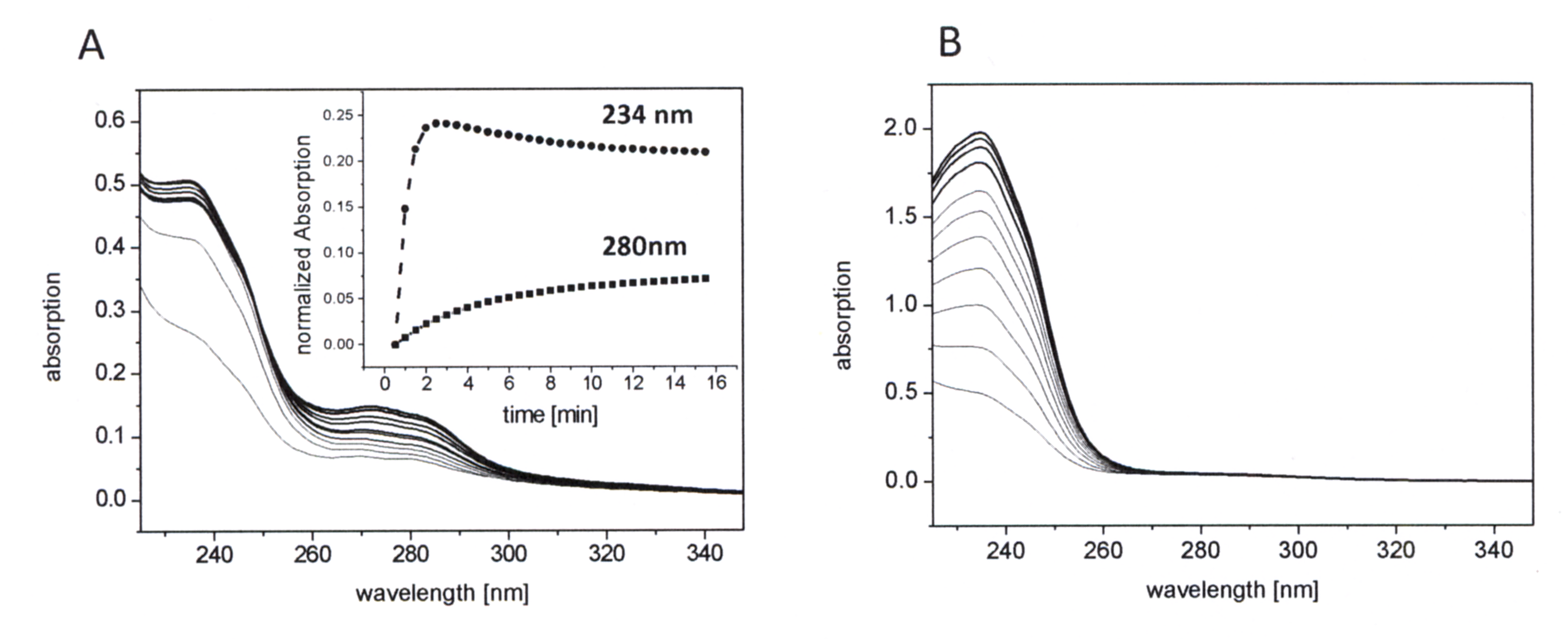

Supplement: Figure S3 — Analysis of time dependent changes in the absorption properties of products formed from incubations of FoxLOX with (A) 18∶3(n-3) and (B) 18∶2(n-6), respectively. Product spectra were recorded for 16 min at different time points using the scanning kinetic technique. (TIF) [file pone.0064919.s003.tif]

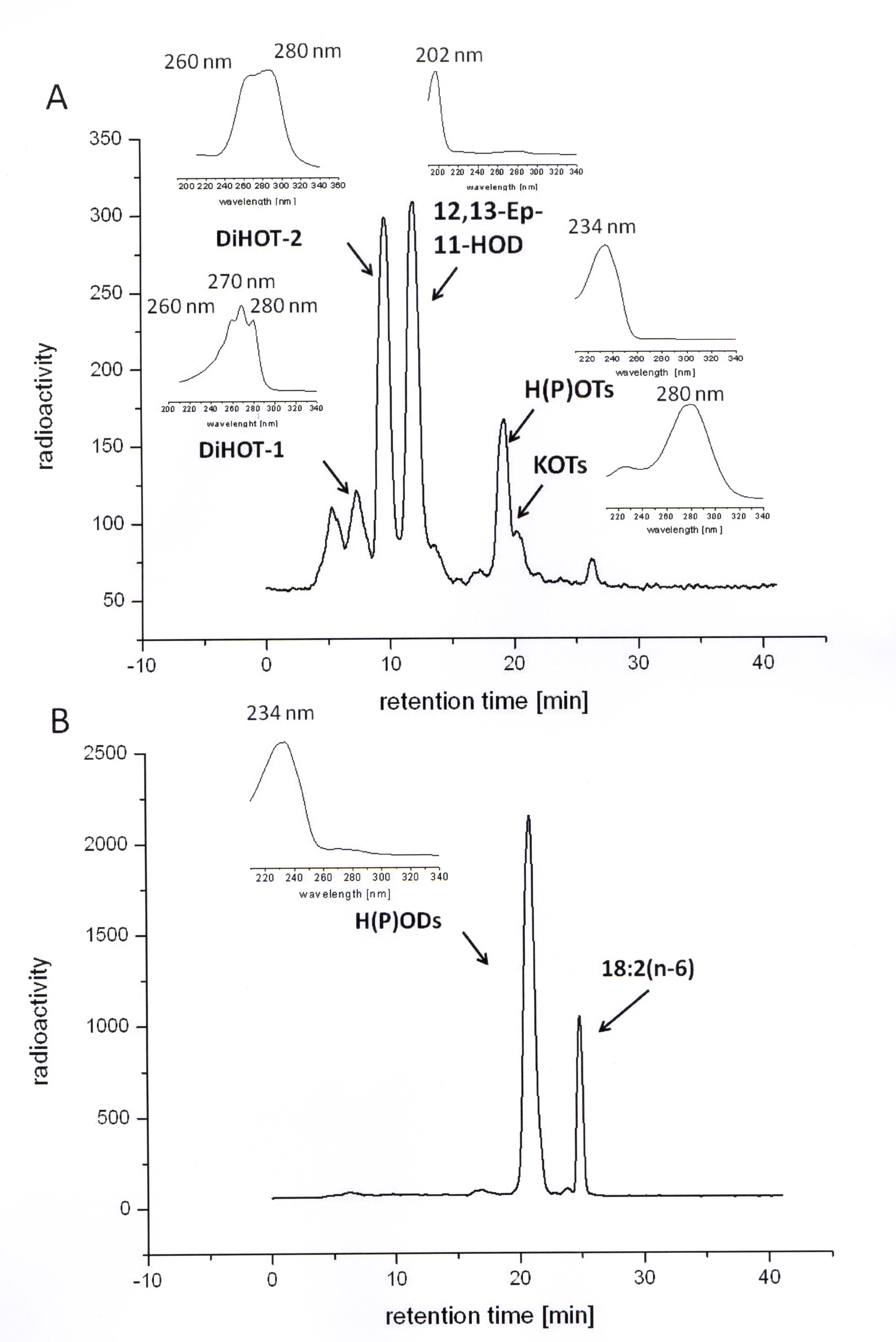

Supplement: Figure S4 — Analysis of the product profile formed from incubations of (A) [1-14C]-18∶3(n-3) and (B) [1-14C]-18∶2(n-6) with FoxLOX. A representative radio-HPLC product profile that was derived from incubations of FoxLOX with [1-14C]-18∶3(n-3) and [1-14C]-18∶2(n-6) is shown in (A) and (B), respectively. The analysis was carried out on a RP-HPLC-system that was coupled to diode-array- and a radio-detector. While chromatogram displays the signals recorded by the radio-detector, the insets show the absorption spectra of the respective products that were record by the diode-array detector. It should be emphasized that the products, shown in this figure, were only assigned based on their retention time, spectral properties and in accordance to data from different other independent experiments (cf. figure S5). Please also note that due to different levels of DiHOT-derivatives detected in this experiment, apparent differences in the respective absorption properties are caused. Therefore the DiHOT-2 product was only tentatively assigned, because its high amounts may led to absorption signals reaching the saturation of the diode array detector leading suboptimal spectral resolution. (TIF) [file pone.0064919.s004.tif]

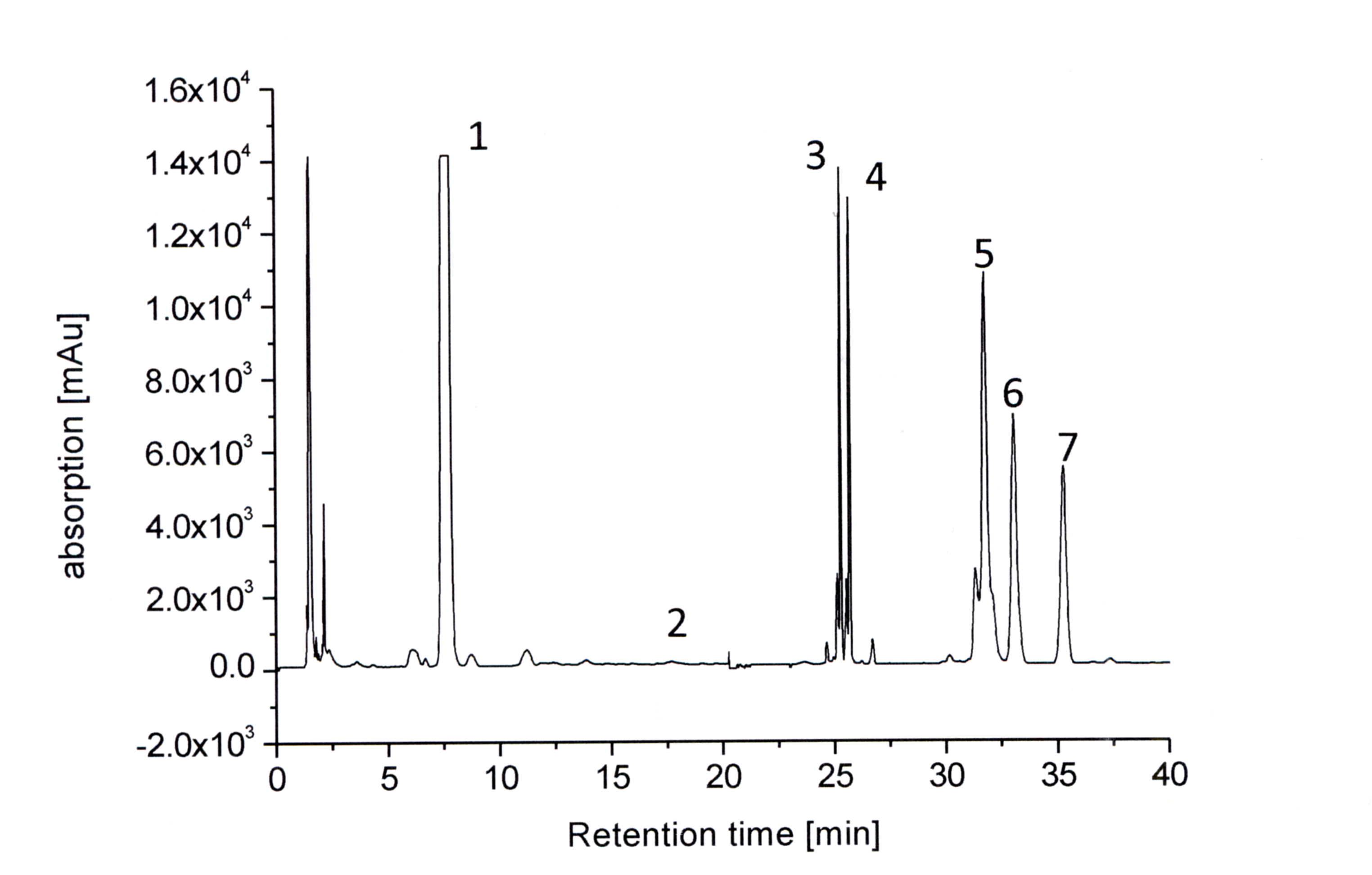

Supplement: Figure S5 — Separation of reduced and methyl-esterified products of 18∶3(n-3) oxygenation by FoxLOX. Elution was accomplished at a rate of 2 mL/min with 0.7% 2-propanol/hexane (0–20 min; UV detection at 210 nm) followed by 5% 2-propanol/hexane (20–40 min; UV detection at 270 nm). Materials forming peaks 1–7 were collected and identified by GC-MS as follows: 13-HOT methyl ester (peak 1), methyl 12S,13S-11-hydroxy-9(Z),15(Z)-octadecadienoate (peak 2; ratio threo/erythro forms, 93∶7), methyl 9-methoxy-16-hydroxy-10,12,14-octadecatrienoate (diastereomers in 1∶1 ratio; peaks 3 and 4), methyl 15,16-dihydroxy-9,11,13-octadecatrienoate (peak 5; ratio erythro/threo forms, 81∶19), and methyl 9,16-dihydroxy-10,12,14-octadecatrienoate (diastereomers in 1∶1 ratio; peaks 6 and 7). (TIF) [file pone.0064919.s005.tif]

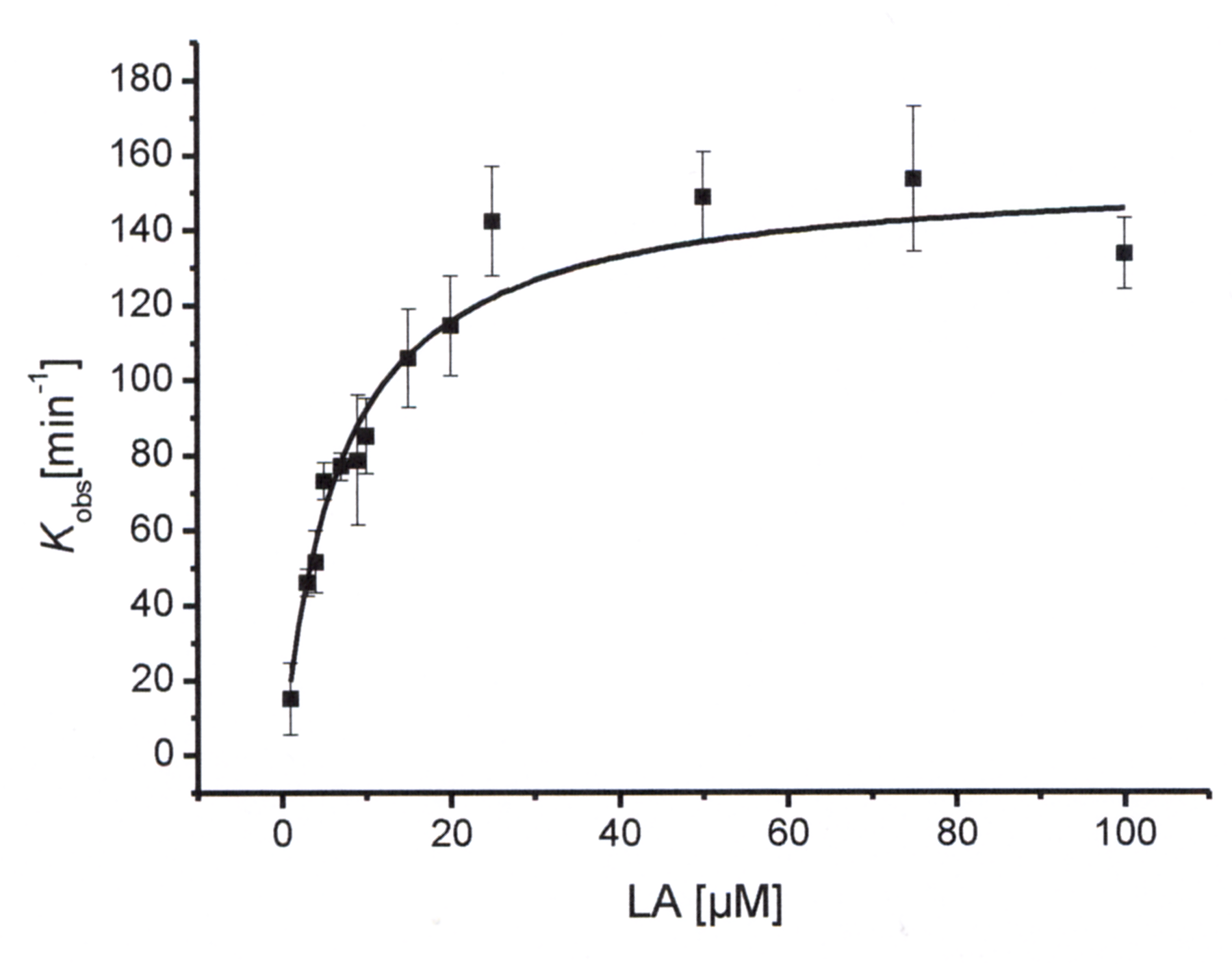

Supplement: Figure S6 — Analysis of steady state kinetics from incubations of FoxLOX with different concentrations of 18∶2(n-6). Initial time dependent changes at 234 nm were determined at different substrate concentrations. The results are presented as mean values ± standard deviation derived from triplicate measurements. Data points were plotted and fitted by employing the Michaelis-Menten equation to derive the kinetic constants. (TIF) [file pone.0064919.s006.tif]
